# Study of Coumarin-Resveratrol Hybrids as Potent Antioxidant Compounds

**DOI:** 10.3390/molecules20023290

**Published:** 2015-02-16

**Authors:** Maria J. Matos, Francisco Mura, Saleta Vazquez-Rodriguez, Fernanda Borges, Lourdes Santana, Eugenio Uriarte, Claudio Olea-Azar

**Affiliations:** 1 CIQUP/Departamento de Química e Bioquímica, Faculdade de Ciências, Universidade do Porto, 4169-007 Porto, Portugal; E-Mails: svre77@gmail.com (S.V.-R.); mfernandamborges@gmail.com (F.B.); 2 Departamento de Química Orgánica, Facultad de Farmacia, Universidad de Santiago de Compostela, 15782 Santiago de Compostela, Spain; E-Mails: lourdes.santana@usc.es (L.S.); eugenio.uriarte@usc.es (E.U.); 3 Departamento de Química Inorgánica y Analítica, Facultad de Ciencias Químicas y Farmacéuticas, Universidad de Chile, Casilla 233 Santiago, Chile; E-Mail: franciscomura@gmail.com

**Keywords:** hydroxylated 3-phenylcoumarins, antioxidant assays, electrochemical study, inhibition of ROS, ESR, ADME properties

## Abstract

In the present work we synthesized a selected series of hydroxylated 3-phenylcoumarins **5**–**8**, with the aim of evaluating in detail their antioxidant properties. From an in depth study of the antioxidant capacity data (ORAC-FL, ESR, CV and ROS inhibition) it was concluded that these derivatives are very good antioxidants, with very interesting profiles in all the performed assays. The study of the effect of the number and position of the hydroxyl groups on the antioxidant activity was the principal aim of this study. In particular, 7-hydroxy-3-(3'-hydroxy)phenylcoumarin (**8**) proved to be the most active and effective antioxidant of the selected series in four of the performed assays (ORAC-FL = 11.8, capacity of scavenging hydroxyl radicals = 54%, Trolox index = 2.33 and AI_30_ index = 0.18). However, the presence of two hydroxyl groups on this molecule did not increase greatly the activity profile. Theoretical evaluation of ADME properties of all the derivatives was also carried out. All the compounds can act as potential candidates for preventing or minimizing the free radical overproduction in oxidative-stress related diseases. These preliminary findings encourage us to perform a future structural optimization of this family of compounds.

## 1. Introduction

Polyphenolic compounds are one of the major families of plant metabolites. These compounds are bioactive substances that have one or more aromatic rings in their structure, bearing one or more hydroxyl groups. This family of compounds acts as antioxidants and thereby protect from degenerative diseases in which reactive oxygen species (ROS) are involved [1]. In fact, the overproduction of free radicals have been related to cellular membrane, protein, RNA and DNA damage, and indirectly with aging and oxidative-stress related diseases like cancer, cardiovascular, inflammatory and neurodegenerative pathologies [2]. The properties of phenolic compounds are related to their chemical structure, which confers stability to the secondary free radical formed from the antioxidant reaction product with a free radical [3]. Therefore, the research and characterization of new bioactive phenolic substances from the diet has been intensified in the last years, either for the development of nutraceuticals or new drugs [4].

Hydroxycoumarins are phenolic compounds which act as potent metal chelators and free radical scavengers [5,6,7,8]. Hydroxylated 3-arylcoumarins are a family of polyphenolic compounds, which are known to possess anti-inflammatory, anti-thrombotic, enzyme inhibitory and antioxidant properties [9,10,11,12]. In particular, our research group has been studying the potential of differently substituted 3-arylcoumarins as antioxidant agents [13,14,15,16,17]. In these studies it was concluded that the position and number of different electron donator groups (hydroxyl, methoxy or methyl) in the scaffold are structural factors contributing to modulate the antioxidant capacity of those compounds. The studied coumarins exhibited, in general, excellent antioxidant activities, which depended on the position and number of hydroxyl groups [13,14,15,16,17].

Resveratrol, structurally 3,4',5-trihydroxystilbene, is a natural phenolic component of *Vitis vinifera* L. and other spermatophyte species, produced in response to exterior or interior damage [18]. Resveratrol shows a large number of pharmacological activities, including anti-inflammatory, antioxidant, anticancer and cardioprotective properties [18]. Some of these properties, such as antioxidant activity, are coincident with those of the hydroxycoumarins.

**Scheme 1:**
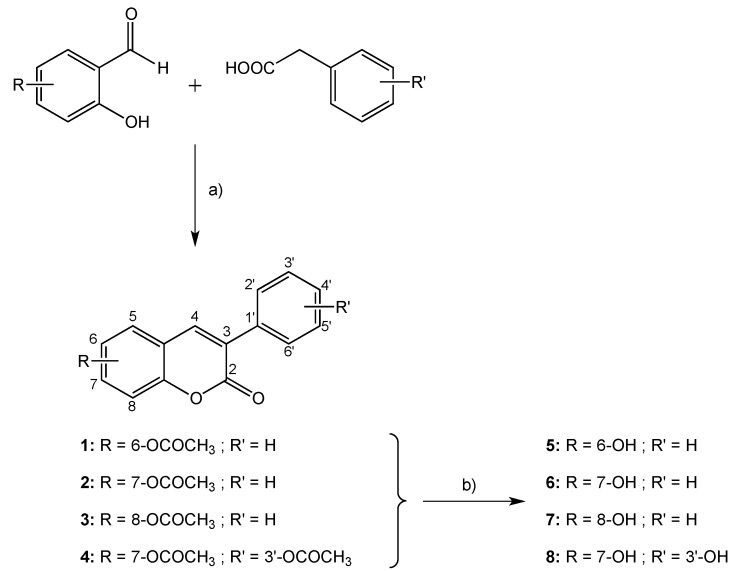
Synthetic methodology to obtained hydroxy-3-phenylcoumarins **5**–**8**.

Taking into account this background, we proposed to study the antioxidant capacity of a selected series of hydroxylated 3-phenylcoumarins (coumarin-resveratrol hybrids—Scheme 1), with one hydroxyl group at positions 6, 7 or 8 (compounds **5**, **6** and **7**, respectively), or two hydroxyl groups at positions 7 and 3' (compound **8**). It is our aim to study the effect of the position and the number of hydroxyl groups on the antioxidant capacity of 3-arylcoumarins. A detailed antioxidant study of the molecules was carried out, using different assays and methodologies. Since the formation of ROS is presented in several biochemical processes and pathologies, these compounds could be valuable as multitarget molecules against several diseases.

## 2. Results and Discussion

### 2.1. Chemistry

In order to obtain the desired final products, coumarin derivatives **5**–**8** were efficiently synthesized according to the protocol outlined in Scheme 1 [15,19,20,21,22,23,24]. The synthetic methodology was carried out in two steps, and is briefly described as follows: (a) synthesis of acetoxy-3-phenylcoumarins **1**–**4** and (b) hydrolysis of the previous compounds to afford the final hydroxy-3-phenylcoumarins **5**–**8**. The general reaction conditions of the synthesized compounds are described in the Experimental Section. The acetoxy-3-phenylcoumarins were efficiently synthesized in 85%–92% yield by a Perkin-Oglialoro condensation [25], treating the appropriate *ortho*-hydroxybenzaldehyde with the adequate phenylacetic acid, in the presence of potassium acetate and acetic anhydride. Hydroxyl derivatives were obtained in with 90%–94% yield from the abovementioned acetoxy-substituted precursors by acidic hydrolysis, using hydrochloric acid in the presence of methanol. 

*Reagents and conditions:* (**a**) CH_3_CO_2_K, Ac_2_O, reflux, 16 h; (**b**) HCl 2N, MeOH, reflux, 4 h.

### 2.2. Antioxidant Capacity Assays

The main objective of this study was to assess in detail the antioxidant capacity of the hydroxy-3-phenylcoumarins **5**–**8** in order to compare the profiles of these compounds as antioxidant agents. To achieve this goal, the oxidation potentials of compounds **5**–**8** were determined by CV [26]. This parameter gave information not only for evaluating the antioxidant potentials of the compounds, but also for understanding their reaction mechanisms [26]. The evaluation of the antioxidant activity of the studied compounds towards different types of ROS: peroxyl, hydroxyl, alkoxyl and superoxide radicals, was also performed [27]. ORAC, ESR and CV were the techniques used to obtain the desired results.

#### 2.2.1. Cyclic Voltammetry (CV)

The first assay performed to better understand the antioxidant potential of the studied compounds was the evaluation of oxidative potentials by CV. Anodic peak potential (Epa) values for compounds **5**–**8** are reported in Table 1, and demonstrated that the coumarin derivatives present a variety of oxidation potentials depending on their substitution pattern. 

**Table 1:** Oxidant potentials recorded for the studied compounds.

Compounds	Epa (V) ^a^
**5**	0.762
**6**	0.640
**7**	0.652
**8**	0.720

^a^ Epa: anodic peak potential. First oxidation peak potential at scan rate of 2000 mV/s. All experiments were carried out in triplicate. The data are expressed as means ± SD.

In the experimental conditions, a single oxidation process (peak I) was observed. In general, it has been proposed that the charge transfer process at peak I corresponded to the oxidation of the hydroxyl substituent. The compounds with lowest oxidation potential were compounds **6** (Epa = 0.640 V) and **7** (Epa = 0.652 V). It is known that the antioxidant capacity is conceivably related to the electrochemical behaviour, being indicative that a low oxidation potential corresponds to a high antioxidant power. 

**Figure 1:**
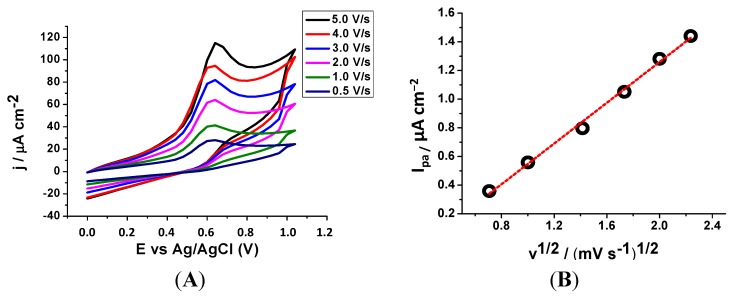
(**A**) Cyclic voltammogram for 1 mmol/L of compound **6**, in DMSO/75 mmol/L phosphate (pH 7.4) buffer 40/60 media at a GCE, for *v* = 0.5–5 mV/s. (**B**) Graphic of the anodic peak current *versus* the root of the scanning rate.

However, this does not present a linear relationship. The low oxidation potential values are only indicative of a good antioxidant profile. Therefore, compound **6** proved to be the molecule with best profile of the series. As an example, the cyclic voltammogram of compound **6** is illustrated in Figure 1. It was recorded at a GCE, in a pH 7.4, containing 1 mmol/L of compound **6**, for several scan rates (*v*). Analysing the results, it was observed by CV that all the studied coumarin derivatives showed the same oxidation pattern; a single oxidation signal, near 0.7 V, corresponding to an irreversible type of reaction (Figure 1A). From the analysis of the anodic peak current (I_pa_) *versus* *v*^1/2^ (Figure 1B), a straight line with r^2^ > 0.99 was obtained, indicating that the electrode process was controlled by diffusion. 

#### 2.2.2. ORAC-Fluorescein (ORAC-FL)

The ORAC-fluorescein (ORAC-FL) assay, that can assess the scavenging ability of coumarins against peroxyl radicals in competition with a fluorescent sensor, was performed and the results expressed as ORAC-FL index are presented in Table 2 [28,29]. In this study, AAPH was used as the peroxyl radical source [30].

**Table 2:** ORAC-FL values calculated for the studied and reference compounds.

Compounds	ORAC-FL Index ^a^
**5**	11.0 ± 2.3
**6**	9.6 ± 1.3
**7**	5.7 ± 0.6
**8**	11.8 ± 1.4
Trolox	1
Quercetin	7.28 ^b^
6,7-Dihydroxy-4-methylcoumarin	3.3 ^c^

^a^ ORAC-FL studies were carried out in 75 mmol/L sodium phosphate buffer (pH 7.4) with coumarin solutions in methanol (range of concentration between 0.3 and 2 μM); ^b^ Reference [26]; ^c^ Reference [27]. All experiments were carried out in triplicate. The data are expressed as means ± SD.

Analyzing the ORAC-FL results, compounds **5** and **8** proved to be the best candidates of the series (ORAC-FL = 11.0 and 11.8, respectively). Nevertheless, compound **6** is also considered a good candidate, presenting an ORAC-FL index of 9.6, significantly higher than quercetin, used as reference compound. The results obtained are better than the ORAC-FL value of simple coumarins, such as the 6,7-dihydroxy-4-methylcoumarin, studied by our group (ORAC-FL = 3.3). An enhanced activity of compound **8**, comparing to compound **6**, was found against this radical, proving that the presence of two hydroxyl groups in the molecules could slightly improve the antioxidant capacity. It was observed that the derivative with the lowest rate is the one bearing the hydroxyl group at position 8 (compound **7**). This may be due to the formation of an intramolecular hydrogen bond with the oxygen of the lactone, depleting the availability of the hydrogen.

**Figure 2:**
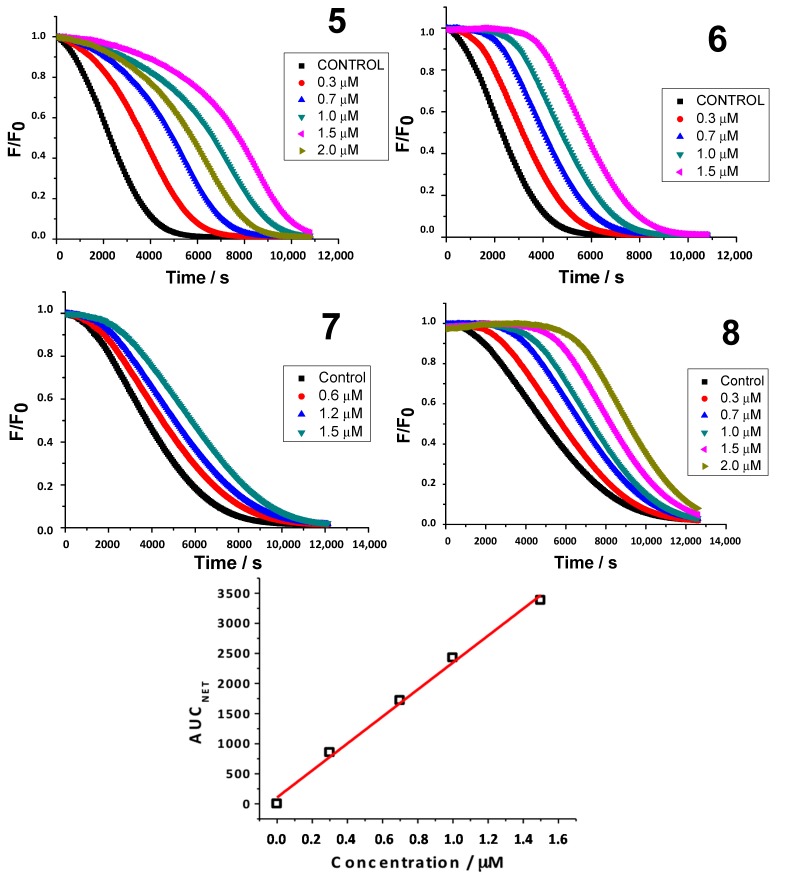
Kinetic profiles of FL consumption AAPH-mediated in presence of compounds **5**–**8**. F_0_ is the fluorescence in absence of the compound and F the fluorescence in presence of the compound. Last graphic: AUC_NET_ *versus* concentration of compound **6**.

The consumption of FL is commonly inhibited, even by antioxidants of low reactivity, throughout kinetic profiles. Figure 2 illustrates the results of AAPH-mediated FL oxidation in the absence and presence of an increasing concentration of compounds **5**–**8**, and demonstrates that the AUC_NET_ of the kinetic profiles for these compounds were linearly related to the concentration of the additive. Of the studied series, compounds **6** and **8** presented an induction time, indicating that these coumarins display better activity to protect the sensor of the peroxyl radical than compounds **5** and **7**, which showed no induction time. In addition, the presence of an induction time observed in the case of compound **6** is accompanied by the fact that the slopes are similar for all concentrations. This indicates that the antioxidant was completely consumed before the fluorescence decay, due to the attack of free radicals remaining on the sensor.

#### 2.2.3. Electron Spin Resonance (ESR)

In order to test the ability of the selected compounds to scavenge hydroxyl radicals a non-catalytic and competitive-type Fenton system was examined [31]. The percentage of scavenging of hydroxyl radicals by the studied compounds was measured and is summarized in Table 3. Antioxidant capacity against alkoxyl radicals, generated by photolysis of AAPH in aqueous medium, was also determined by spin trapping methodology, and the results expressed as Trolox indexes are summarized in Table 3.

**Table 3:** Percentage of scavenging of hydroxyl radicals and Trolox index calculated for compounds **5**–**8**.

Compounds	% Scavenging of Hydroxyl Radicals ^a^	Trolox Index
**5**	24.7 ± 6.3	2.28 ± 0.05
**6**	44.7 ± 1.2	2.32 ± 0.05
**7**	10.9 ± 1.1	1.23 ± 0.01
**8**	51.4 ± 4.5	2.33 ± 0.05
Trolox	-	1

^a^ Scavenging activity of hydroxyl radicals effect was calculated as follows: [(A_0_ − A_x_)/A_0_] × 100, where A_x_ and A_0_ are the double-integral ESR for the first line of samples in the presence and absence of test compounds, respectively. All experiments were carried out in triplicate. The data are expressed as means ± SD.

**Figure 3:**
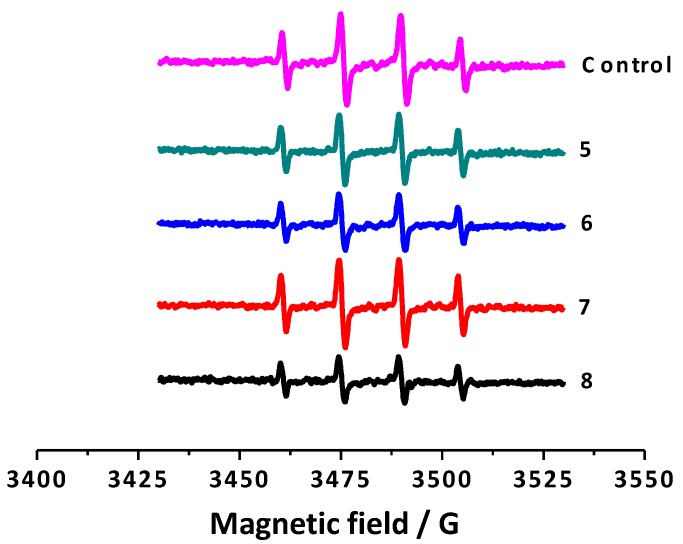
ESR spectra of the control and 3-phenylcoumarins **5**–**8**.

To study the antioxidant capacity of all the synthesized coumarins towards hydroxyl radicals in depth, a non-catalytic and competitive type Fenton system was set up, employing DMPO as a spin trap. The percentage of scavenging of the studied compounds was measured and is summarized in Table 3. In this assay, DMPO reacts with hydroxyl radicals to generate the spin resonance signal, which was quantified by ESR [32]. The ESR spectrum illustrates four hyperfine lines due to the DMPO-OH adduct formation. In particular, ESR spectrum obtained to the control (DMPO + *N*,*N*-dimethylformamide + NaOH + H_2_O_2_) presents four hyperfine lines illustrated in magenta (Figure 3). Coumarin derivatives competed with DMPO for hydroxyl radicals, diminishing the ESR signal, as represented in Figure 3 for compounds **5**–**8**.

As expected, the intensity of the spectra decreased when the coumarin derivatives were added to the system. This type of response was observed for all derivatives, reflecting different percentages of hydroxyl radical scavenging activity (Table 3). Compound **8** proved to be the best compound of the studied series, presenting the highest hydroxyl radical scavenging capacity (51.4%). This value could be due to the presence of two hydroxyl groups in its structure. Compounds **6** and **8**, bearing a hydroxyl group at position 7, proved to be the molecules with highest capacity to scavenge hydroxyl radicals. An enhanced activity of compound **8**, compared to compound **6**, was found against this radical, proving that the presence of two hydroxyl groups in the molecules could slightly improve the antioxidant capacity.

#### 2.2.4. Antioxidant Capacity against Alkoxyl Radicals by ESR 

**Figure 4:**
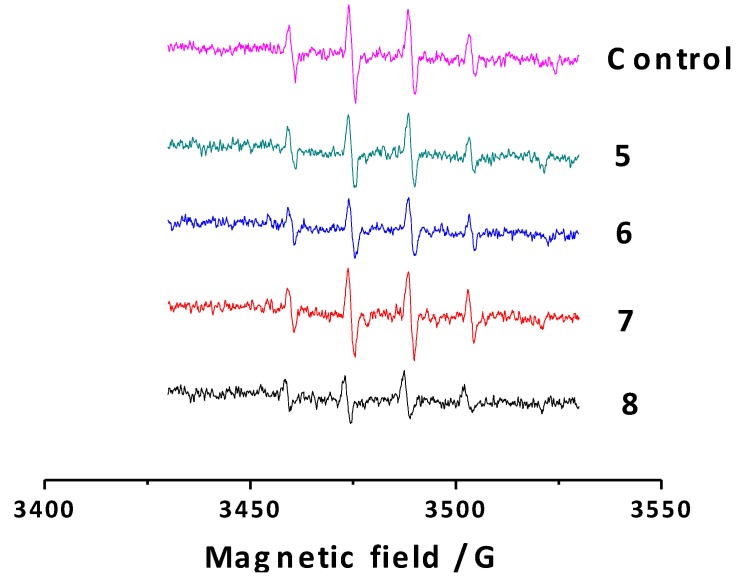
Spectrogram of the adduct formed between AAPH-derived radical by photolysis and DMPO. G-value: 2.0023. Microwave frequency: 9.81 GHz, Modulation amplitude: 0.95 G, Time constant: 81.92 ms, Conversion time: 40.96 ms. Simple LW = 0.598.

Antioxidant capacity against alkoxyl radicals generated by photolysis of AAPH in aqueous medium was monitored by spin trapping methodology [33,34,35,36]. The antioxidant capacity was considered proportional to the fall of the signal height compared to the control, in absence of antioxidant (Figure 4). All the results were contrasted with Trolox, being the Trolox index proportional to the antioxidant capacity against alkoxyl radicals (Table 3).

The results described in Table 3 showed the same tendency observed to the ORAC-FL, in which compounds **5**, **6** and **8** presented a similar antioxidant capacity, bigger than compound **7**. The second hydroxyl group in the 3-phenyl moiety (compound **8**) seems to have not significantly improve the antioxidant capacity against alkoxyl radicals.

#### 2.2.5. Antioxidant Activity against Superoxide Radicals by CV

**Figure 5:**
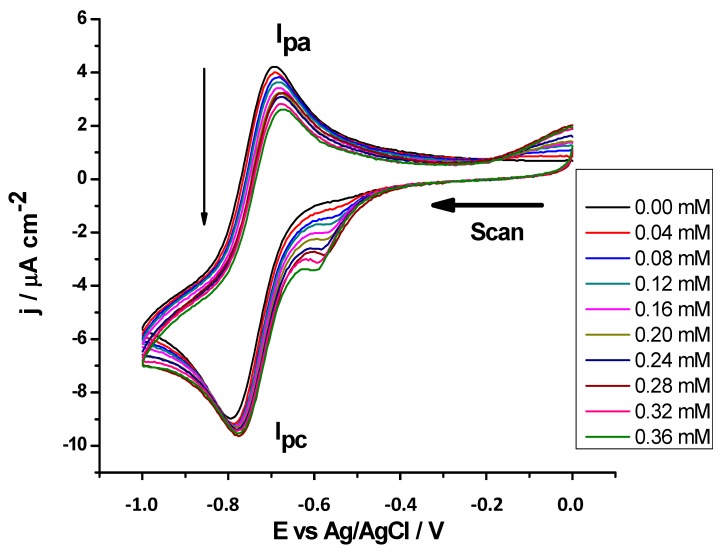
Cyclic voltammograms of superoxide radical at different concentrations of compound **6** in DMSO + TBAP 0.1 M, on GC (working electrode) *versus* Ag/AgCl, at room temperature, with scan rate of 30 mV/s.

In order to investigate the antioxidant activity of the coumarins against the superoxide anion radical (O_2_**^−^**) generated electrochemically [37,38], a stock solution of the coumarins was added gradually to the solution, leading to a decrease of O_2_**^−^** I_pa_, while the intensity of O_2_ cathodic current was not significantly modified, as shown in Figure 5. The oxidation potential peaks of the studied coumarins did not interfere with the superoxide radical couple signal, which appeared at more negative potentials. For each antioxidant compound, a series of I_pa_ values was determined from the CVs recorded for increasing antioxidant concentrations. All antioxidant substrates exhibited a similar effect upon the O_2_ reduction. At higher concentrations of some antioxidant substrates, a not-well defined reduction peak was observed at a potential more positive than the oxygen reduction peak.

By analogy, with the inhibiting concentration of antioxidant (IC), the antioxidant index values expressed by the substrate concentration needed to consume 30% (AI_30_ index) by the concentration in millimoles of antioxidant substrate needed to consume superoxide radicals was determined as revealed by a current decrease to 30% of the initial anodic current (I_pa_°) (AI_30_ = (I_pa_° − I_pa_^s^)/I_pa_° = 0.30). With this antioxidant characterization, the lower the AI_30_ values are, the more antioxidant capacity the substrate has against superoxide [39,40]. Recorded data is listed in Table 4. 

**Table 4:** AI_30_ index calculated for compounds **5**–**8**.

Compounds	AI_30_/mM
**5**	0.19 ± 0.02
**6**	0.23 ± 0.02
**7**	0.23 ± 0.04
**8**	0.18 ± 0.04
Trolox	0.24 ± 0.02

All experiments were carried out in triplicate. The data are expressed as means ± SD.

**Figure 6:**
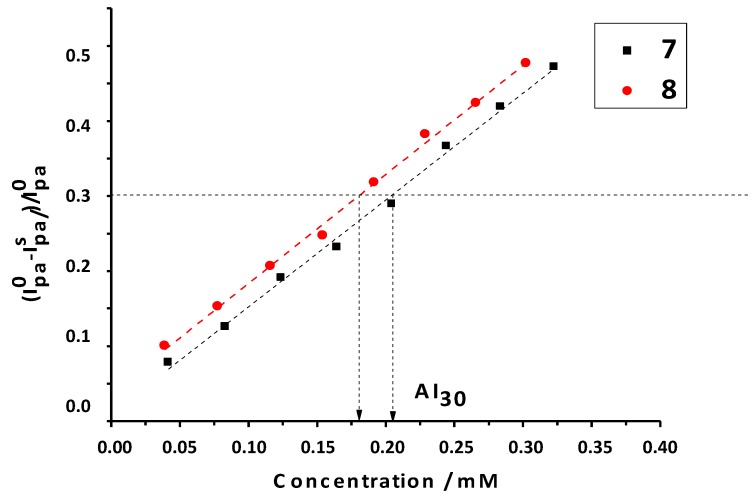
Graphs of decreasing anodic peak current between the control (I_pa_°) and the substrate (I_pa_^s^) against increasing concentrations of substrate, in this case, compounds **7** and **8**; AI_30_ is the concentration of inhibition at 30% of superoxide radical.

It was observed that the best superoxide scavenger of this series was once again compound **8**, which had the lowest AI_30_. However, as in some of the above-described assays, none of the studied compounds presented significant differences in their AI_30_ values (Figure 6).

#### 2.2.6. Inhibition of ROS

**Figure 7:**
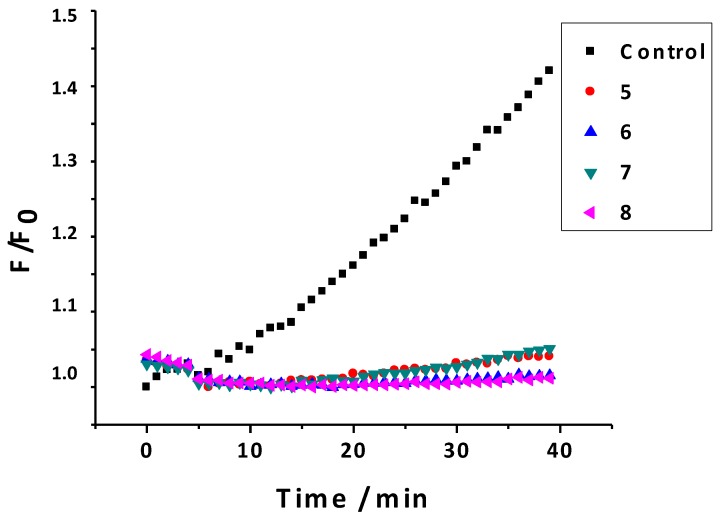
Graphic of ROS inhibition (control and compounds **5**–**8** at 10 μM).

An assay on cell models (macrophages from the strain RAW 264.7) was performed to better understand the antioxidant potential of the studied compounds in a biological environment [41]. This assay allowed determining the amount of ROS formed after an oxidation process. This process is particularly sensitive to the detection of H_2_O_2_ and peroxyl radicals. The percentage of disappearance of fluorescence in presence of menadione (50 μM) and different concentrations of the 3-phenylcoumarins (1, 10 and 100 μM) was evaluated, and the results of this study are presented in Table 5 (1 μM) and Figure 7 (10 μM). 

**Table 5:** Percentage of disappearance of fluorescence in presence of menadione (50 μM) and 3-phenylcoumarins **5**–**8** (1 μM), (*p* < 0.05).

Compounds	% Disappearance of Fluorescence
**5**	92.57 ± 0.09
**6**	92.52 ± 0.03
**7**	42.88 ± 0.30
**8**	88.69 ± 0.08
Trolox	1.48 ± 0.01

All experiments were carried out in triplicate. The data are expressed as means ± SD.

The quenching capacity was biologically studied using radicals generated by stimulation of oxidative stress due to the inclusion of menadione in a cellular model. Menadione is capable of generating a wide concentration responsible for the occurrence of free radical fluorescence due to oxidation of the sensor.

Subsequently, with the addition of coumarin at concentrations of 1, 10 and 100 μM, a decrease in fluorescence was observed, indicating that the coumarin effectively entered the cell and protected it from oxidation (Figure 7). In this assay, the smaller is the slope, and the less is the fluorescence, better antioxidant capacity the compounds present. At 10 μM, the effect of all the studied coumarins was less than 10% of appearance of fluorescence (Figure 7). At lower concentrations (1 μM), three of the four studied compounds (compounds **5**, **6** and **8**) still presented less than 10% of appearance of fluorescence. However, compound **7** presented 57% of appearance of fluorescence. 

### 2.3. ADME Theoretical Properties Calculation 

To better correlate the drug-like properties of the studied compounds the lipophilicity, expressed as the octanol/water partition coefficient and herein called logP, as well as other theoretical calculations such as the topological polar surface area (TPSA), the number of hydrogen acceptors and the number of hydrogen bond donors were calculated using the Molinspiration property program [42]. TPSA is a commonly used medicinal chemistry metric for the optimization of a drug’s ability to permeate cells and achieve the desired target. The obtained *in silico* results are summarized in Table 6 [42].

**Table 6:** Theoretical structural properties of the 3-phenylcoumarins **5**–**8**. ^a^

Compd.	logP	TPSA (Å^2^)	*n-*OH Acceptors	*n-*OHNH Donors	Volume (Å^3^)
**5**	3.23	50.44	3	1	208.01
**6**	3.23	50.44	3	1	208.01
**7**	3.23	50.44	3	1	208.01
**8**	2.73	70.67	4	2	216.03

^a^ TPSA, topological polar surface area; *n*-OH, number of hydrogen acceptors; *n*-OHNH, number of hydrogen bond donors. The data was determined with Molinspiration calculation software [42].

From the data obtained for the theoretical evaluation of the ADME properties, it can be noticed that none of the compounds **5**–**8** break any of the Lipinski rule of five, making them promising leads for drug candidates. TPSA and logP values are compatible with those described as a predictive indicator of the drug capacity of membrane penetration [42,43].

## 3. Experimental Section

### 3.1. Synthesis

General procedure for the synthesis of acetoxy-3-phenylcoumarins **1**–**8**. Compounds **1**–**4** were synthesized under anhydrous conditions, using materials previously dried at 60 °C for at least 12 h and at 300 °C during few minutes immediately before use. A solution containing anhydrous potassium acetate (CH_3_CO_2_K, 2.94 mmol), the conveniently substituted phenylacetic acid derivative (1.67 mmol) and the corresponding 2-hydroxybenzaldehyde (1.67 mmol) in acetic anhydride (Ac_2_O, 1.2 mL) was refluxed (138 °C) for 16 h. The reaction mixture was cooled, neutralized with 10% aqueous sodium bicarbonate (NaHCO_3_), and extracted (3 × 30 mL) with ethyl acetate (EtOAc). The organic layers were combined, washed with distilled water, dried with anhydrous sodium sulfate (Na_2_SO_4_), and evaporated under reduced pressure. The product was purified by recrystallization in ethanol (EtOH) and dried, to afford the desired compound. Hydroxylated coumarins **5**–**8** were obtained by hydrolysis of their acetoxylated counterparts **1**–**4**. In general, the appropriate acetoxylated coumarin was mixed with 2 N aqueous hydrochloric acid (HCl) and methanol (MeOH) and refluxed (100 °C) with stirring during 4 h. The resulting reaction mixtures were cooled in an ice-bath and the reaction products, obtained as solids, were filtered, washed with cold distilled water, and dried under vacuum, to afford the desired compound. Characterization of the compounds was previously reported [14,19,20,21,22,23,24].

### 3.2. Antioxidant Assays

#### 3.2.1. Oxidation Potential Determination by Cyclic Voltammetry (CV)

Cyclic voltammetry (CV) was carried out using a Metrohm instrument (Riverview, FL, USA) with a 797 VA stand convertor and a 797 VA processor, in DMSO/75 mM phosphate (pH 7.4) buffer 40/60 DMSO/(~1.0 Å, ~10.3 M) at room temperature with KCl (~0.1 M), using a three-electrode cell. A glassy carbon electrode (GCE) was used as the working electrode, a platinum wire as the auxiliary electrode, and saturated calomel (Ag/AgCl) as the reference electrode. The assays were performed containing 1 mmol/L of each studied compound, for several scan rates (*v* = 0.5–5 mV/s).

#### 3.2.2. Oxygen Radical Antioxidant Capacity-Fluorescein (ORAC-FL)

The oxygen radical absorbance capacity (ORAC-FL) studies were carried out on a Synergy™ HT multidetection microplate reader from Bio-Tek Instruments, Inc. (Winooski, VT, USA), using white polystyrene 96-well plates, purchased from Nunc (Roskilde, Denmark) [27,28,30,44,45,46]. Fluorescence was read from the top, with an excitation wavelength of 485/20 nm and an emission filter of 528/20 nm. The plate reader was controlled by Gen 5 software. The reaction was carried out in 75 mM sodium phosphate buffer (pH 7.4), and 200 μL final volume. Fluorescence (FL; 40 nM, final concentration) and coumarin solutions in methanol (range of concentration between 0.3 and 2 μM) were placed in each well of the 96-well plate. The mixture was pre-incubated for 15 min at 37 °C, before rapidly adding the 2,2'-azo-bis(2-amidinopropane)dihydrochloride (AAPH) solution (18 mM, final concentration). The microplate was immediately placed in the reader and automatically shaken prior to each reading. The fluorescence was recorded every 1 min for 120 min. A blank with FL and AAPH using methanol instead of the antioxidant solution was used in each assay. Five calibration solutions using Trolox^®^ (0.5–2.5 μM) as the antioxidant were also used in each assay. The inhibition capacity was expressed as ORAC-FL values and it was quantified by integration of the area under the fluorescence decay curve (AUC). All reaction mixtures were prepared in triplicate and at least three independent assays were performed for each sample. The AUC was calculated integrating the decay of the fluorescence, where F0 is the initial fluorescence read at 0 min and F is the fluorescence read at time. The net AUC corresponding to the sample was calculated by subtracting the AUC corresponding to the blank. Data processing was performed using Origin Pro 8 SR2 (Origin Lab Corporation, Northampton, MA, USA).

#### 3.2.3. Hydroxyl Radical Scavenging Assay Using Electron Spin Resonance (ESR) 

Reactivity of all the hydroxy-3-phenylcoumarin derivatives against the hydroxyl radical was investigated using the non-catalytic Fenton type method. Electron spin resonance (ESR) spectra were recorded in the X band (9.7 GHz) using an ECS 106 spectrometer (Bruker, Coventry, UK) with a rectangular cavity and 50 kHz field modulation, equipped with a high-sensitivity resonator at room temperature. Spectrometer conditions were: microwave frequency 9.81 GHz, microwave power 20 mW, modulation amplitude 0.91 G, receiver gain 59 db, time constant 81.92 ms and conversion time 40.96 ms [46]. The scavenging activity of each derivative was estimated by comparing the 5,5-dimethyl-1-pyrroline-*N*-oxide (DMPO-OH) adduct signals in the antioxidant-radical reaction mixture and the control reaction at the same reaction time, and was expressed as scavenging percent of hydroxyl radical. To prepare the samples, 150 μL of *N*,*N*-dimethylformamide (DMF) and 50 μL of NaOH (3 mM) were mixed, followed by the addition of 50 μL of DMPO spin trap (30 mM final concentration) and finally 50 μL of hydrogen peroxide 30%. The mixture was put in an ESR cell and the spectrum was recorded after five minutes of reaction. All the compounds were studied at 4 mM final concentration (300 μL final volume).

#### 3.2.4. Determination of Alcoxyl Radicals Generated by Photolysis of AAPH Assay Using Electron Spin Resonance (ESR)

Antioxidant capacity against alcoxyl radicals generated by photolysis of AAPH in aqueous medium was monitored by spin trapping methodology (ORAC-like assay). The most popular spin trap for oxygen-centered free radicals is the previously described DMPO. The photolysis of AAPH, at 392 nm, had been identified to generate alcoxyl radicals, observing a splitting pattern of 4 lines with hyperfine coupling constant of a_N_ = 14.27 G and a_H_ = 14.67 G, for the adduct DMPO/RO^•^ [33,34,35]. Moreover, it was not found the appearance of methyl radical/DMPO adduct, product of the reaction of hydroxyl radical and DMSO-*d*_6_ [34]. This was an evidence that pattern of lines is due to DMPO/RO^•^, not DMPO/HO^•^. To achieve this goal, coumarins were prepared in DMSO at concentration of 0.1 mM, DMPO was prepared in 0.1 M phosphate buffer pH 7.4 at concentration of 200 mM, and AAPH was prepared in buffer medium at concentration of 20 mM. The antioxidant capacity was considered proportional to the decrease of signal height compared to the control, in absence of antioxidant. 

#### 3.2.5. Superoxide Antioxidant Assay Using Cyclic Voltammetry (CV)

Superoxide anion radical was generated one electron reduction of the atmospheric molecular oxygen in DMSO of analytical grade (Sigma-Aldrich, St. Louis, MO, USA), with 0.1 M tetrabutylammonium perchlorate (TBAP) as supporting electrolyte. Then, the voltammetric performance of the compounds was studied. Coumarins were added incrementally to the *in situ* generated radical and the resultant behavior was recorded. The concentration of the coumarins in the electrochemical cell was in the range of 0.0 to 0.4 mM. The scan rate was kept at 50 mV/s and the potential window was −1.0–0.0 V [47,48]. The atmospheric solubility of oxygen in DMSO was 2.1 mM. The antioxidant activity was assessed from the change in the cathodic current of the voltammograms in absence and present of the derivatives, using pertinent mathematical formulations. The relative decrease in the intensity signal was expressed as (I_pa_° − I_pa_^s^)/I_pa_°; where I_pa_° is the current peak in the oxidative scan in absence of substrate, and I_pa_^s^ is the current peak in the oxidative scan in presence of substrate. CV measurements were performed in a Metrohm instrument with a 694VA stand convertor and a 693VA processor, at room temperature, using a three-electrode cell. A GC electrode presenting an area of 0.03 cm^2^ was used as the working electrode. The electrode surface was polished to a mirror finish with alumina powder (0.3 and 0.05 LM) before use and after each measurement. Platinum wire was used as auxiliary electrode and silver-silver chloride (Ag/AgCl, 3 M KCl) of Metrohm Company with a plastic tip was used as a reference electrode. 

### 3.3. Statistical Data Analysis

Statistical analyses were conducted using GraphPad Prism 5 software (GraphPad Software, Inc., San Diego, CA, USA). The data are expressed as means ± SD. The experimental data were analyzed by one-way analysis of variance (ANOVA), and differences between groups were assessed using Tukey’s post-test. The level of significance was set at *p* < 0.05, and all experiments were replicated 3 times.

### 3.4. Inhibition of Radical Oxygen Species (ROS)

A variety of reductive enzymes such as NADPH-cytochrome P450 reductase and NADH microsomal-ubiquinone oxidoreductase (complex I) are able to metabolize quinones by reductive reactions via electron [41,49]. The resulting semiquinone radical can enter a redox cycle, if oxygen is present, regenerating the starting quinone and produce ROS. In the present assay, the used quinone source was menadione (2-methylnaftoquinone) 50 µM/well, and the fluorescent sensor was dichlorodihydrofluoscein diacetate (DCFH_2_-DA) 20 µM/well, which is a non-fluorescent compound that penetrated by diffusion into the cell, and was hydrolysed to 2',7'-dichlorodihydrofluorecein (DCFH_2_) inside the cell, making it susceptible to be oxidized by ROS, giving the fluorescent compound 2',7'-dichlorofluorescein (DCF) [41,49]. Measuring the fluorescence emitted from the DCF at 530 nm, after being excited at 495 nm, allowed determining the amount of ROS formed after the oxidation process. This process is particularly sensitive to detection of H_2_O_2_ and peroxyl radicals. The cell models used in this study were macrophages from the strain RAW 264.7. The medium used for the assay was a phosphate buffer solution 0.01 M (0.138 M NaCl; 2.7 mM KCl; pH 7.4). The studied coumarins were prepared in DMSO/buffer (no more tan 1% DMSO) to final concentrations of 1, 10 and 100 µM. The macrophage cells were diluted in a phosphate buffer solution until concentrations of 50.000 cell/mL. 

### 3.5. Theoretical Evaluation of ADME Properties

The ADME properties of the studied compounds were calculated using the Molinspiration property program. LogP was calculated by the methodology developed by Molinspiration as a sum of fragment-based contributions and correction factors [42]. Topological Polar Surface Area (TPSA) was calculated based on the methodology published by Ertl *et al.* as a sum of fragment contributions. Oxygen and nitrogen centered polar fragments were considered. PSA had been shown to be a very good descriptor characterizing drug absorption, including intestinal absorption, bioavailability, Caco-2 permeability and blood-brain barrier penetration. Method for calculation of molecule volume developed at Molinspiration was based on group contributions. These had been obtained by fitting sum of fragment contributions to “real” 3D volume for a training set of about twelve thousand, mostly drug-like molecules. 3D molecular geometries for a training set were fully optimized by the semiempirical AM1 method.

## 4. Conclusions

Oxidation potentials of all the studied hydroxylated 3-phenylcoumarins were determined, with compounds **6** and **7** giving the lowest oxidation potentials, associated to a higher antioxidant activity within the series. Through the ORAC-FL test, derivative **8**, bearing two hydroxyl groups on the scaffold, presented the highest value (11.8), similar to compound **5** (11.0). In the spin-trapping assays, the highest percentage of scavenging of hydroxyl radicals (51.4%) and alkoxyl radicals (trolox index = 2.33) also corresponded to compound **8**. However, these values are similar to those of compound **6** (scavenging of hydroxyl radicals = 44.7% and Trolox index = 2.32). An enhanced activity of compound **8** (compared to compound **6**) was found, particularly against peroxyl and hydroxyl radicals. The AI_30_ index of compound **8** was also the best one in the series (0.18). Finally, in the biological assay, it was observed that all coumarins decreased ROS generation in an oxidative stress situation due to the metabolism of menadione. As main conclusion, the position of the hydroxyl group on the benzene ring of the coumarin scaffold is in general critical for the antioxidant activity, even though all the derivatives presented a good profile. Also, in general, the increase of a hydroxyl group in the 3-phenyl ring did not improve significantly the antioxidant profile of the derivatives. Therefore these preliminary findings have encouraged us to perform a future structural optimization of this kind of compounds.
